# Bidirectional triplet exciton transfer between silicon nanocrystals and perylene†

**DOI:** 10.1039/d1sc00311a

**Published:** 2021-04-05

**Authors:** Tingting Huang, Timothy T. Koh, Joseph Schwan, Tiffany T.-T. Tran, Pan Xia, Kefu Wang, Lorenzo Mangolini, Ming L. Tang, Sean T. Roberts

**Affiliations:** Department of Chemistry, University of California Riverside Riverside CA 92521 USA mltang@ucr.edu; Department of Mechanical Engineering, University of California Riverside Riverside CA 92521 USA lmangolini@engr.ucr.edu; Materials Science & Engineering Program, University of California Riverside Riverside CA 92521 USA; Department of Chemistry, The University of Texas at Austin Austin TX 78712 USA roberts@cm.utexas.edu; Center for Dynamics and Control of Materials, The University of Texas at Austin Austin TX 78712 USA

## Abstract

Hybrid materials comprised of inorganic quantum dots functionalized with small-molecule organic chromophores have emerged as promising materials for reshaping light's energy content. Quantum dots in these structures can serve as light harvesting antennas that absorb photons and pass their energy to molecules bound to their surface in the form of spin-triplet excitons. Energy passed in this manner can fuel upconversion schemes that use triplet fusion to convert infrared light into visible emission. Likewise, triplet excitons passed in the opposite direction, from molecules to quantum dots, can enable solar cells that use singlet fission to circumvent the Shockley–Queisser limit. Silicon QDs represent a key target for these hybrid materials due to silicon's biocompatibility and preeminence within the solar energy market. However, while triplet transfer from silicon QDs to molecules has been observed, no reports to date have shown evidence of energy moving in the reverse direction. Here, we address this gap by creating silicon QDs functionalized with perylene chromophores that exhibit bidirectional triplet exciton transfer. Using transient absorption, we find triplet transfer from silicon to perylene takes place over 4.2 μs while energy transfer in the reverse direction occurs two orders of magnitude faster, on a 22 ns timescale. To demonstrate this system's utility, we use it to create a photon upconversion system that generates blue emission at 475 nm using photons with wavelengths as long as 730 nm. Our work shows formation of covalent linkages between silicon and organic molecules can provide sufficient electronic coupling to allow efficient bidirectional triplet exchange, enabling new technologies for photon conversion.

## Abstract 2

We demonstrate that silicon quantum dots can exchange spin triplet excitons with molecules covalently attached to their surface. Such hybrid materials can enable systems that upconvert incoherent far-red light into the visible spectral range.

## Graphical abstract

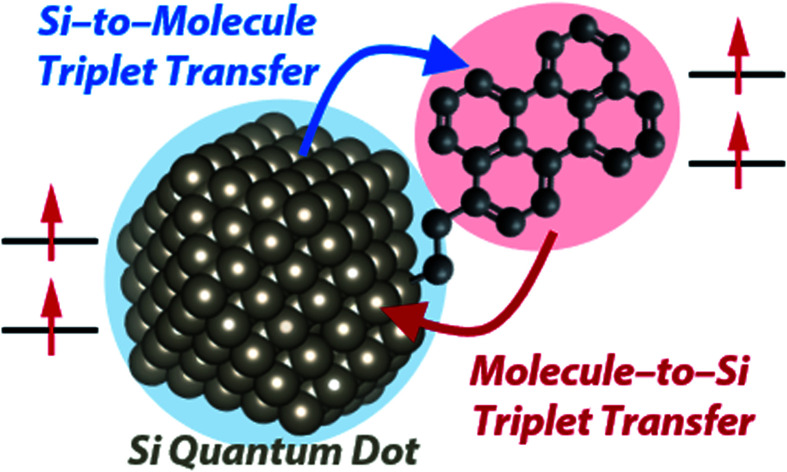

Hybrid materials comprised of inorganic quantum dots (QDs) interfaced with small-molecule organic chromophores have emerged as a promising platform for materials that convert near-infrared radiation into the visible spectral range.^1–3^ In these structures, QDs act as light-harvesting antennas, absorbing long-wavelength photons and passing their energy to organic molecules bound to their surface in the form of spin-triplet excitons. These excitons can then be transferred into a surrounding medium, typically a solution or thin film, where pairs of them can fuse to form a bright spin-singlet state that can emit a short-wavelength photon.^4–8^ Due to the long lifetime of molecular triplet excitons, which can range from several microseconds to milliseconds, these materials can operate at low photon flux, enabling their integration into light-harvesting systems that operate under solar flux^9,10^ and limiting heat dissipation during their use in biological applications, such as phototherapy,^11,12^ live-cell imaging,^13,14^ and optogenetics.^15^ These hybrid materials can also be used to study interfacial energy transfer processes fundamental to the operation of solar cells that use triplet fusion's inverse process, singlet fission, to enhance their performance.^9,16–21^ The simplest design for a cell of this type is one that interfaces a singlet fission material directly in line with a back-contacted semiconductor solar cell.^22–24^ In these structures, the singlet fission material acts as a light sensitizer that captures high-energy photons and uses their energy to generate pairs of triplet excitons that can be passed to the semiconductor to produce photocurrent. As molecules can be readily attached to QDs *via* a variety of chemical tethers, these materials allow detailed study of how the structure of the organic:inorganic interface impacts the ability of triplet excitons to move from one material to the other.

For both triplet fusion-based light upconversion and singlet fission-based light harvesting, silicon represents a key material of interest. While several upconversion systems have been derived using QDs containing toxic elements, such as Cd^5,7,25^ or Pb,^6,8,26,27^ Si QDs are nontoxic, making them attractive for biological applications.^28^ Silicon also dominates the solar energy market, accounting for ∼90% of solar power production,^29,30^ making Si:organic interfaces that readily transmit triplet excitons a key design target for singlet fission-based solar cells.^18,19,22^ Previously, we have shown triplet exciton transfer from Si QDs to surface-bound anthracene molecules can power a photon upconversion system that operates with 7% efficiency.^31^ However, the inverse energy transfer process that is key for singlet fission devices, triplet exciton transfer from surface-bound molecules to Si, was not observed in our prior work.

In this report, we address triplet exciton transfer from molecules to Si by demonstrating a hybrid Si QD:perylene system wherein photoexcitation of the Si QD establishes a spin-triplet exciton population that exists in a dynamic equilibrium between the QD and perylene molecules bound to its surface. While such exciton cycling has been reported for other QD:molecule systems,^32–34^ our work represents the first observation of this behavior in Si QD based systems. Using nanosecond transient absorption spectroscopy, we find triplet exciton transfer from Si to perylene takes place on a 4.2 μs timescale while energy transfer in the reverse direction occurs more than two orders of magnitude faster, on a 22 ns timescale. We attribute this difference in energy transfer rates to differences in the exciton density of states between perylene molecules and Si QDs. To demonstrate the utility of triplet excitons produced by this system for photon conversion applications, we have constructed a photon upconversion system by interfacing perylene-functionalized Si QDs with a complementary perylene-based triplet fusion annihilator. We find this system performs well, upconverting radiation with a wavelength as long as 730 nm into blue light centered near 475 nm. Under 532 nm illumination, the system upconverts light with an efficiency of 1.5% under incident light fluxes as low as 80 mW cm^−2^. This performance is comparable to that recently demonstrated using the same perylene annihilator coupled with a Pd-porphyrin light absorber.^35^ Our work demonstrates that the introduction of short, chemical linkers between molecules and Si can enable triplet exciton exchange between these materials for the design of new systems for both photon upconversion and light harvesting.

## Results & discussion

**Scheme 1:**
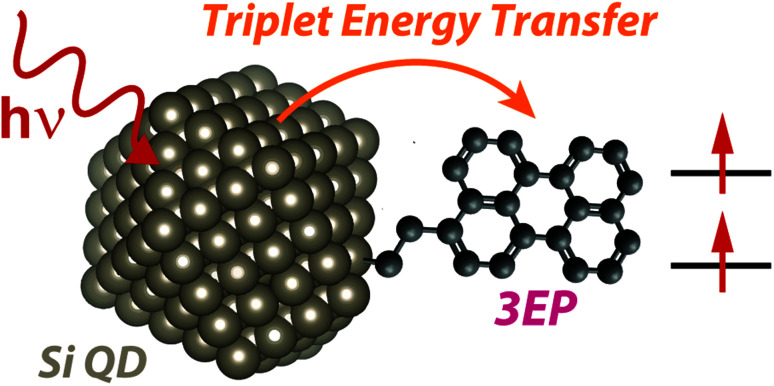


**Fig. 1:**
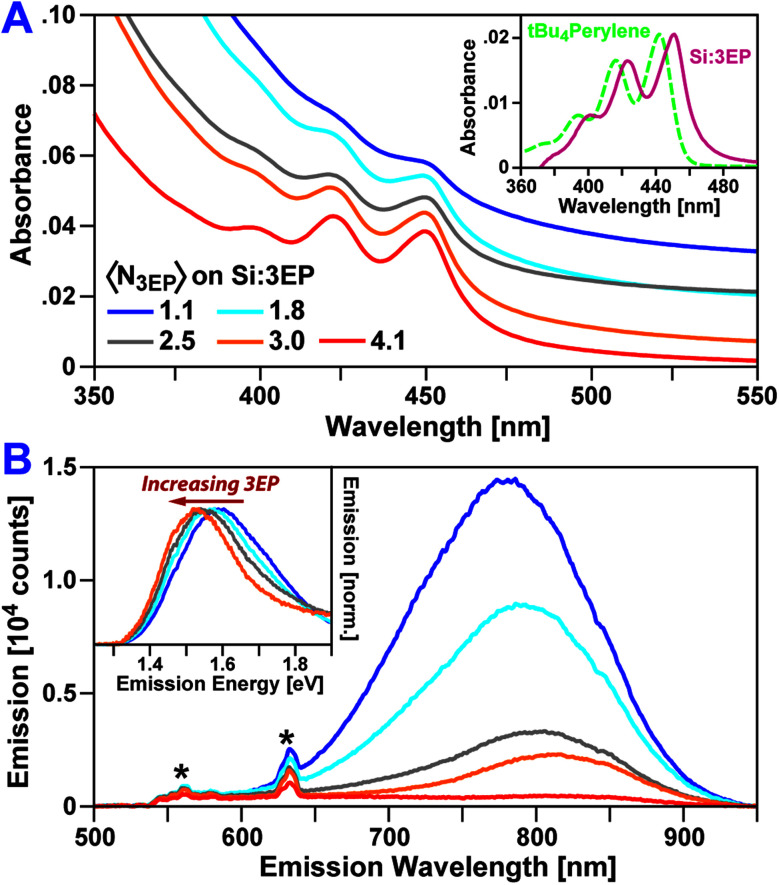
(A) Absorption spectra of Si QDs functionalized with increasing numbers of 3EP molecules. QDs are dispersed in toluene. (Inset) 3EP absorption features are bathochromically shifted by 53 meV from a perylene reference compound (*t*Bu_4_perylene). (B) Corresponding emission spectra of 3EP-functionalized Si QDs (*λ*_Ex_ = 532 nm). As the number of 3EP molecules bound to a Si QD increases, QD emission is quenched. Features designated with a “*” correspond to Raman scattering peaks. (Inset) As Si QD emission is quenched by 3EP attachment, a bathchromic shift and narrowing of the residual QD emission is seen.

The hybrid Si QD:perylene system whose structure and dynamics we report below is illustrated in Scheme 1. Hydrogen-terminated Si nanocrystals were synthesized from silane gas by flowing it through a nonthermal plasma^36,37^ (see ESI† for details). These nanocrystals are quantum confined and exhibit a broad emission peaked at 780 nm (1.59 eV) following hydrosilylation with octadecene (Fig. 1). This energy lies above that of perylene's lowest-energy triplet state (1.53 eV)^38–42^ but below that of its lowest excited singlet state (2.6 eV), suggesting triplet energy transfer from Si to perylene is the only viable energy transfer pathway between these materials.

Initially, we attempted to functionalize Si QDs with perylene *via* a thermal hydrosilylation protocol we have used previously to prepare Si QDs with mixed monolayers of 9-ethylanthracene (9EA) and octadecane.^31^ However, we found reactions using 3-vinylperylene (3VP) in place of an anthracene precursor resulted in a hybrid organo-silicon material that did not perform photon upconversion. Rather, this material gives a broad, featureless photoluminescence spanning from 550–900 nm, suggesting formation of an inhomogeneous mixture of nano-silicon and molecular species (Fig. S3†). Fortunately, by lowering the synthesis temperature from 180 °C to 60 °C, and using 2,2′-azobis(2-methylpropionitrile) (AIBN) as a radical initiator, we found mixtures of 3VP and octadecene (ODE) gave colloidally stable Si QDs with both 3-ethylperylene (3EP) and octadecane solubilizing groups covalently bound to their surface (see ESI† for detailed synthetic methodology). We label these hybrid Si QD:perylene complexes as Si:3EP. We note that a mixture of 3EP and octadecane (ODA) is present on the surface of Si:3EP QDs even though ODA is not denoted by our naming scheme.

Fig. 1 plots absorption and emission spectra of Si:3EP prepared using different ratios of 3VP to ODE during Si QD functionalization. 3VP surface attachment is signaled by the appearance of a distinct vibronic progression starting at 450 nm that stems from 3EP's S_1_ state. We find increasing the 3VP-to-ODE mole ratio used during functionalization from 0.02 to 0.5% 3EP increases the strength of 3EP absorption features relative to those of the Si QDs following washing to remove unbound ligand, indicating progressive binding of larger quantities of 3EP to Si. As the average number of 3EP molecules attached to Si increases, vibronic features associated with 3EP's electronic absorption spectra grow in strength but do not display any appreciable shifts in their spectral position or linewidth (Table 1), indicating 3EP molecules bound to Si are spatially separated. Interestingly, these vibronic features are noticeably redshifted by 53 meV from the absorption spectrum of a *tert*-butyl substituted perylene derivative, 2,5,8,11-tetra-*tert*-butylperylene (*t*Bu_4_perylene), dispersed in solution (Fig. 1A, inset). A similar shift in electronic absorption spectra of aromatic molecules attached to Si QDs was seen for 9EA-functionalized QDs^31^ and suggests some degree of electronic coupling between 3EP molecules and the Si QDs to which they are bonded.

**Table 1:** Absorption and emission properties of Si:3EP samples whose optical spectra appear in Fig. 1. These samples were synthesized *via* low-temperature, radical-driven hydrosilylation using a precursor solution containing different mol ratios of 3VP and ODE (3VP/ODE). For each Si QD sample, *λ*_MAX_ 3EP denotes the absorption maxima of surface-bound 3EP, *λ*_MAX_ Si PL the Si QD emission maximum, and Si PL QY the Si QD emission quantum yield using rhodamine 6G as a standard (*λ*_Ex_ = 532 nm)

Sample	3VP/ODE (%)	*λ*_MAX_ 3EP (nm)	*λ*_MAX_ Si PL (nm)	Si PL QY (%)
Si:ODA	0	N/A	774	8.6
Si:3EP	0.02	449	775	10.5
Si:3EP	0.05	449	781	6.2
Si:3EP	0.10	449	784	5.5
Si:3EP	0.20	450	792	2.6
Si:3EP	0.50	451	—	1.0

3EP functionalization also quenches Si QD photoluminescence (PL) (Fig. 1B), with the magnitude of this effect increasing as a QD binds greater numbers of 3EP molecules. This quenching is highly suggestive of energy transfer from Si to surface-bound 3EP. We also observe a progressive bathochromic shift of the Si QD emission peak and a narrowing of the QD emission linewidth as 3EP binds (Fig. 1B, inset). This shift and narrowing is qualitatively similar to that observed in Si:9EA hybrid complexes^31^ where it was assigned to preferential quenching of small Si QDs with wide bandgaps that matched 9EA's triplet energy. Analogous Si emission lineshape changes for Si:3EP suggest a similar effect is at play in this system.

**Fig. 2:**
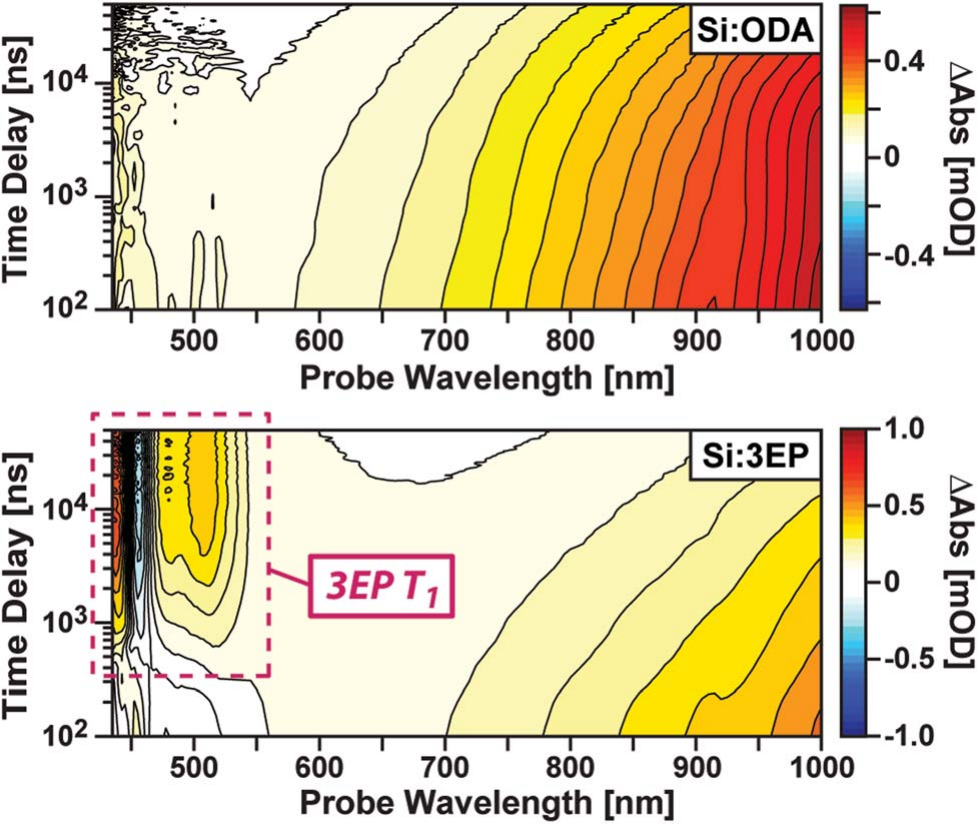
Transient absorption spectra of functionalized Si QDs. (Top) Spectra of Si:ODA show a broad induced absorption that arises from photoexcited carriers. (Bottom) Spectra of Si:3EP show an accelerated decay of Si induced absorption bands that reveals spectral features indicative of 3EP's T_1_ state (maroon dashed).

To identify the origin of the emission quenching we used nanosecond transient absorption to characterize the excited-state dynamics of Si:ODA and Si:3EP. Fig. 2 plots transient absorption spectra of Si:ODA recorded following photoexcitation at 532 nm. In accordance with prior reports,^31,43–46^ Si QD photoexcitation leads to the appearance of a broad photoinduced absorption spanning the visible and near-infrared spectral range associated with transitions involving photoexcited carriers. This induced absorption decays over several hundred microseconds with a functional form that cannot be reproduced by a single exponential function, suggesting the presence of multiple quenching channels distributed among the Si QD ensemble. Indeed, we can well reproduce this decay using a model that accounts for different types of characteristic trap sites distributed among the QD ensemble in accordance with a Poisson distribution^31,47^ (Fig. S5†).

Photoexcitation of Si:3EP leads to qualitatively different behavior (Fig. 2, bottom). While the Si photoinduced absorption seen for Si:ODA is observed at early delays, this band decays over the course of a few microseconds concomitant with growth of a negative photobleach peaked at 455 nm and positive induced absorption features at 437, 479 and 508 nm. These features respectively match the spectral position of 3EP's 0–0 ground state absorption peak (Fig. 1A) and prior reports of perylene triplet exciton induced absorption bands.^48,49^ Experiments that use Pt octaethylporphyrin (PtOEP) to sensitize formation of *t*Bu_4_perylene triplet excitons confirm these features arise from 3EP's lowest excited triplet (T_1_) state (Fig. S4B†). As the 3EP triplet grows with the same rate constant measured for loss of Si QD induced absorption features, this indicates 3EP triplet excitons are produced *via* a direct energy transfer process, with no intermediate states populated in between.

**Fig. 3:**
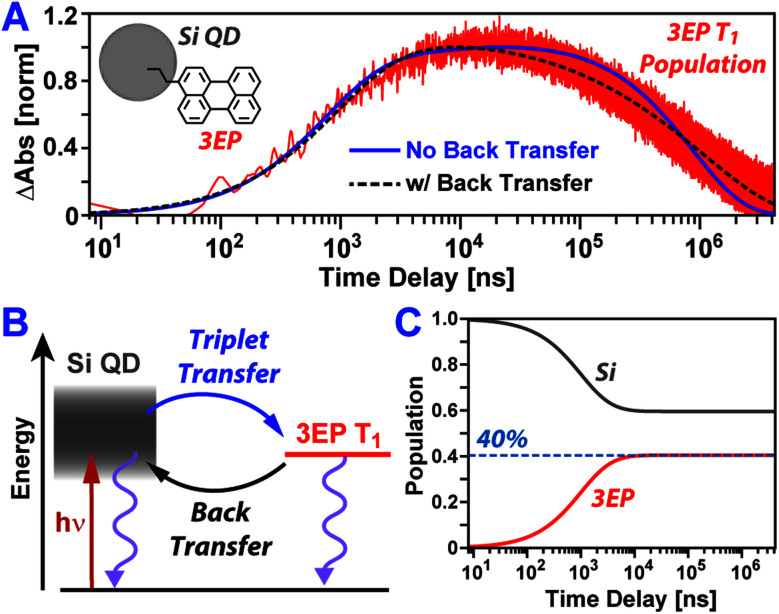
(A) 3EP T_1_ state induced absorption amplitude following photoexcitation of Si:3EP at 532 nm. Plotted alongside the data are fits using the kinetic model shown in (B) that either account for (black dashed) or exclude (blue solid) the possibility of triplet energy back transfer from 3EP to Si. The inclusion of energy back transfer drastically improves the fit to the data. (C) Excited exciton populations of Si and 3EP estimated using best fit values for forward and back triplet energy transfer rates but wherein relaxation processes that return Si QDs and 3EP to their ground state have been removed. If Si QD and 3EP excited populations equilibrate, 40% of the excitations in the system will reside on 3EP.

Fig. 3A plots temporal slices of the Si:3EP dataset taken at a probe wavelength of 505 nm that captures kinetics associated with the growth and decay of the 3EP T_1_ state. This slice highlights that the 3EP T_1_ state develops slowly, appearing with a time constant of 1/*k*_TET_ = 4.2 μs as determined from a kinetic model used to fit the transient absorption dataset (see ESI†). This energy transfer timescale is somewhat surprising given the results of our prior work examining Si QDs functionalized with 9EA (Si:9EA), which found Si-to-9EA triplet energy transfer to occur 280× faster, with a time constant of 1/*k*_TET_ = 15 ns. We note these two rate constants are derived from a Miller–Abrahams hopping model that accounts for how mismatch between the Si QD exciton energy and organic acceptor T_1_ state energy impact triplet energy transfer (*vide infra*). For both Si:3EP and Si:9EA, these triplet transfer rates are those obtained when the Si QD exciton energy is placed in exact energetic resonance with the T_1_ state of the organic acceptor. Hence, a difference in the triplet energy transfer rates for these two systems exists independent of energetic mismatch of Si QD and molecular exciton states. This rate variation also does not stem from differences in the average number of energy acceptors bound to Si QDs in the Si:9EA and Si:3EP samples as we explicitly take this into account using a Poisson distribution when fitting our kinetic model. Estimates of the molar extinction of Si QDs span a range of values,^50,51^ but using these values together with reported extinction spectra for perylene derivatives allows us to estimate between 0.9–4.6 3EP molecules are on average bound to the Si QDs we investigated by transient absorption. Given this range, we do not expect our extracted value for *k*_TET_ for Si:3EP to vary by more than a factor of 2 as a result of uncertainty in this average value, 〈*N*_3EP_〉, which is not large enough to explain the 280× difference in *k*_TET_ between Si:3EP and Si:9EA.

Rather, this transfer rate difference more likely originates from a variation in the electronic coupling between Si and these two distinct energy acceptors. Assuming *k*_TET_ is proportional to the square of this coupling in accordance with Fermi's Golden Rule implies a 17× larger coupling between Si and 9EA relative to Si and 3EP. This stronger coupling could stem from increased wavefunction overlap between 9EA and Si with respect to 3EP. Given our use of identical 2-carbon chains to attach 9EA and 3EP to Si QDs in our prior^31^ and present work, it is likely that both 9EA and 3EP are held at similar distances from the Si surface. However, we note our prior work employed smaller Si QDs with an average exciton energy of 1.67 eV rather than the larger 1.59 eV Si QDs used in the present study. Prior work examining perovskite QDs has suggested increasing exciton confinement can speed the rate of triplet energy transfer to pyrene triplet exciton acceptors by over 3 orders of magnitude by enhancing the spatial leakage of the QD exciton wavefunction out of the surface, increasing its overlap with that of the pyrene energy acceptor.^52^ A similar effect tied to Si QD size could be at play in the systems we have examined.

Shifting our focus to the decay of 3EP's T_1_ state (Fig. 3A, top), we find it is nonexponential. 3EP molecules placed in their T_1_ state return to the ground state with a half-life of ∼600 μs that is ∼7–10× faster than the lifetime reported for perylene monomers in solution, 4–6 ms.^53–55^ This indicates attaching 3EP molecules to Si leads to a shortening of the 3EP T_1_ state lifetime. To determine the origin of this accelerated decay, we have attempted to fit transient absorption spectra of Si:3EP using a kinetic rate model previously used to describe the photoexcited dynamics of Si:9EA.^31^ Key processes included in this model are highlighted in Fig. 3B. Briefly, the model assumes the Si:3EP system is comprised of an ensemble of QDs with differing exciton energies whose distribution is given by the emission lineshape of a similarly prepared Si:ODA sample (Fig. S1B†). As can be seen from this distribution, some Si QDs possess exciton energies that are larger than 3EP's triplet energy while others have exciton energies lower than that of 3EP's T_1_ state. This latter set of QDs face a thermal activation barrier for triplet energy transfer, which we account for by employing a Miller–Abrahams rate expression^56,57^ for Si-to-3EP triplet transfer:1
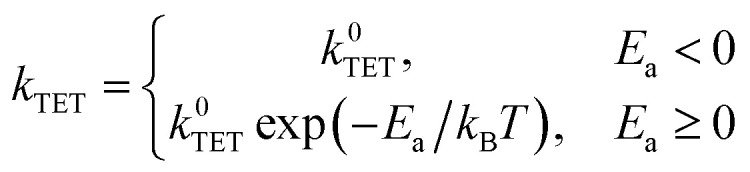
where the activation energy, *E*_a_, is the energy difference between a 3EP molecule's T_1_ state and the exciton energy of the Si QD to which it is bound. We also account for the likely scenario wherein different QDs in the ensemble bind differing numbers of 3EP molecules, which we take to be divided among QDs according to a Poisson distribution whose average value, 〈*N*_3EP_〉, is extracted from ground state absorption spectra. Analytic solutions for populations described by this model are provided in the ESI.†

Fitting this model to the 3EP triplet kinetics reported by transient absorption spectra produces the blue trace Fig. 3A. While this fit captures well the rise of the 3EP triplet population, it does a poor job in reproducing the decay of the population, first underestimating and then overestimating its rate of relaxation as the pump–probe time delay is scanned from tens to hundreds of microseconds. The inability of the present model to fully describe the relaxation of the 3EP triplet exciton population suggests it lacks key energy transfer pathways that contribute to these dynamics.

As the 3EP T_1_ state lies in close energetic resonance with the average exciton energy of the Si QD ensemble, this led us to postulate the model's poor performance in describing 3EP triplet relaxation could stem from formation of a dynamic equilibration of excited population between Si and 3EP, with excitations moving back and forth between these two materials across their interface. Such equilibration has been reported for PbS QDs interfaced with acene energy acceptors,^32,33^ suggesting it could also operate in Si QD-based systems. While we previously concluded that such reverse triplet energy was not needed to model the excited state kinetics of Si:9EA,^31^ we note 9EA molecules examined in that study exhibited a lifetime of only 1.16 μs, a value ∼3 orders of magnitude shorter than the reported lifetime of anthracene monomers in solution.^58,59^ We now believe this accelerated lifetime primarily stems from two sources: (1) incomplete exclusion of oxygen from our prior samples, which can quench 9EA triplets, and (2) a higher density of surface traps on Si particles relative to those we study here. Evidence for this second point is provided by comparing the lifetime of Si QDs used in that study to those reported here, the latter of which are found to exhibit an extended lifetime due to improvements we have made in sample processing and handling conditions (Fig. S5†). These improvements have allowed us to increase the half-life of 9EA triplet excitons bound to Si to values >58 μs (Fig. S6†), leading to upconversion efficiencies of 15%, more than double the value we previously reported. As such, we believe accelerated 9EA triplet quenching prevented establishment of an excited equilibrium between Si and 9EA in our prior work.

Plotted in Fig. 3A is a black-dashed trace that represents a fit to the 3EP T_1_ population dynamics produced by editing our model to allow for triplet energy back transfer, from 3EP to Si. This gives a substantially improved fit to the data as it allows the model to naturally reproduce the nonexponential decay of the 3EP T_1_ population. While addition of an extra parameter to the model would naturally be expected to improve its ability to reproduce experimental data, we note this fit yields a best fit value of 3.2 for 〈*N*_3EP_〉 that falls firmly in the middle of the range of reasonable values we estimate for this parameter (0.9–4.6) based on reported Si QD extinction spectra.^50,51^ The fit also yields a value of 2.3 ms for the intrinsic 3EP triplet lifetime, which agrees well with prior reports^53–55^ and improves our confidence in the model's validity. Examining the extracted time constant for triplet energy back transfer from 3EP to Si, we find it adopts a value of 1/*k*_TEBT_ = 22 ns that is ∼190× faster than the triplet energy transfer rate from Si to 3EP, indicating that for a QD whose exciton energy matches that of the 3EP triplet state, an excited exciton would favor residing in Si. This result makes good sense as Si possesses a large degeneracy of exciton states near its band edge, which entropically favors shifting the exciton population equilibrium towards Si in the absence of an energetic driving force for transfer to 3EP.

Existence of a dynamic equilibrium between 3EP and Si has consequences for Si QD-based photon upconversion systems. For these systems to function, triplets must be able to be extracted *via* triplet transmitting molecules at a QD's surface. Equilibration of population between these molecules and the QD lowers the number of these excitations available for extraction at any given time. While the asymmetry of rates for forward and back triplet transfer within the Si:3EP system we study here may at first glance suggest this equilibrium is skewed heavily towards Si, we note these rates correspond to a case where a Si QD with an exciton energy exactly matched to the triplet state of a 3EP molecule binds only a single 3EP energy acceptor. For the system we examine here, many QDs have larger exciton energies that favor triplet transfer to 3EP and bind several 3EP energy acceptors, potentially skewing the equilibrium towards 3EP. To estimate where the equilibrium triplet position lies for our Si:3EP ensemble, we use our model to compute the time-dependent Si QD and 3EP populations in the absence of pathways that return excited population to the ground state (*k*_Si_ = *k*_3EP_ = 0). Fig. 3C plots these populations and shows that after ∼10 μs, the system reaches an equilibrium where 40% of the excited population has transferred from Si to 3EP.

**Fig. 4:**
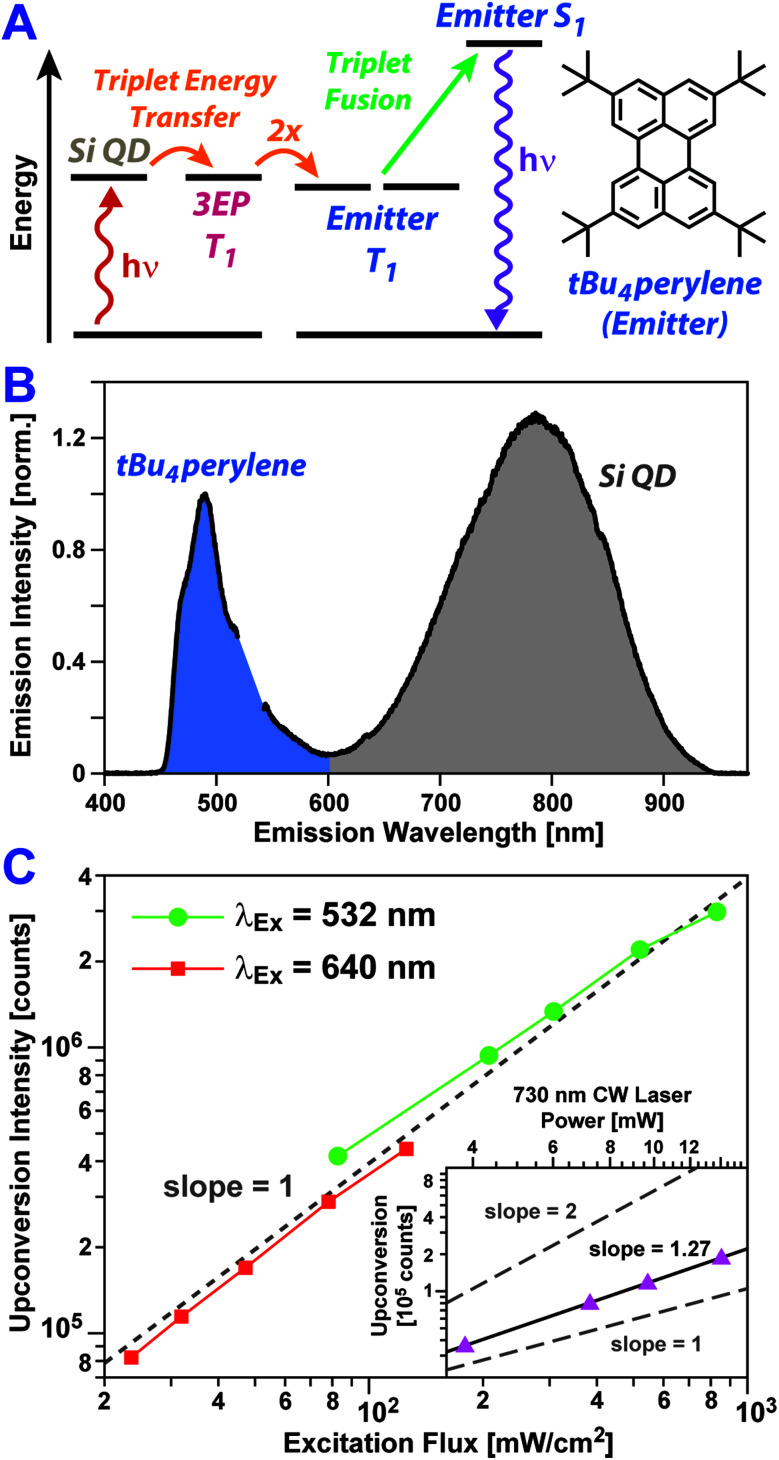
(A) Jablonski diagram showing an energy transfer pathway that produces upconverted emission. (B) Emission spectra of a solution containing Si:3EP and 0.5 mM *t*Bu_4_perylene following photoexcitation of Si at 532 nm. Upconverted emission from *t*Bu_4_perylene (blue shaded) appears in addition to emission from Si QDs (grey shaded). (C) Intensity of upconversion emission from a *t*Bu_4_perylene + Si:3EP solution. Upconversion emission driven by either 532 nm (green circles) or 640 nm light (red squares) scales linearly with the power flux of the excitation source over the power range examined. (Inset) Upconversion emission is seen upon 730 nm excitation of Si, but over the energy range examined, the emission intensity scales with a slope between 1 and 2.

To assess if triplet excitons can be readily extracted from Si:3EP, we have built a photon upconversion system that uses *t*Bu_4_perylene as a light emitter (Fig. 4A). In this system photoexcitation of a Si QD leads to triplet energy transfer to 3EP ligands at its surface that in turn transfer these excitations to diffusing *t*Bu_4_perylene molecules in solution. Pairs of excited *t*Bu_4_perylene molecules can engage in triplet fusion, placing one of the molecules into an emissive singlet exciton state while deexciting the other.

Fig. 4B shows PL spectra of Si:3EP QDs dispersed in a toluene solution containing 0.5 mM *t*Bu_4_perylene. We estimate 〈*N*_3EP_〉 for the Si QDs used for this measurement to fall in the range of 1.2–6.5. Upon excitation of the solution with either green (532 nm) or red (640 nm) light, blue emission stemming from *t*Bu_4_perylene is seen, indicating Si:3EP functions as an effective sensitizer for triplet fusion upconversion. Fig. 4C plots how the upconverted blue *t*Bu_4_perylene PL intensity depends on the energy flux of the excitation source. Under both 532 nm (green circles) and 640 nm excitation (red circles), we observe a linear dependence of the upconverted emission on excitation flux over a range extending from 800 mW cm^−2^ to values as low as 25 mW cm^−2^. A linear dependence on excitation flux, rather than a quadratic dependence, indicates that over this power range triplet excitons transferred to *t*Bu_4_perylene in solution decay primarily *via* triplet fusion.^60,61^ This can only occur if Si-to-*t*Bu_4_perylene triplet transfer *via* surface-bound 3EP molecules is sufficiently efficient as to produce a large concentration of excited *t*Bu_4_perylene molecules, ensuring each can find a partner with which to undergo triplet fusion before it relaxes *via* phosphorescence or intrinsic non-radiative processes. We also note that the binding of 3EP molecules to the surface of Si QDs in the system is key to enabling its function as no light upconversion is observed if Si:3EP is substituted by Si:ODE, which has no perylene bound to its surface. Under 532 nm excitation conditions, we find the system achieves an upconversion efficiency of 1.5%. This value is comparable to that recently reported for an upconversion system employing *t*Bu_4_perylene as the triplet fusion emitter and palladium(ii) *meso*-tetraphenyl-tetrabenzoporphyrin (PdTPTBP) as the light absorber, which displayed upconversion efficiencies ranging from 0.1–7.6% depending on the relative concentration of *t*Bu_4_perylene to PdTPTBP.^35^

Bolstered by these results, we attempted to push the performance of our upconversion system further into the far-red by using a 730 nm laser source to excite Si:3EP. We indeed obtain blue emission upon 730 nm excitation, but over the excitation power range examined, this upconverted emission displayed a dependence on the incident power that lies between a linear and quadratic dependence (slope = 1.27, Fig. 4B, inset). This suggests existence of some inefficiencies that prevent the system from operating in a saturated regime. One potential explanation for this behavior is the decrease of the Si QD's absorption cross-section with increasing wavelength, which would hamper their ability to generate similar exciton populations for identical photon fluxes of green *vs.* red light. Indeed, we find evidence in favor of this explanation as the Si QDs highlighted in Fig. 4B display an upconversion efficiency of 1.5% when excited by 532 nm light in the linear fluence regime whereas this value drops to 0.42% for 640 nm light under similar excitation conditions. This ∼3× difference in upconversion efficiency matches well the extinction ratio of the Si QDs at these two wavelengths. However, the Si QD extinction at 730 nm is only ∼2.5× weaker than it is at 640 nm. This moderate difference suggests reduced extinction does not fully explain the poorer performance of the system under 730 nm excitation conditions.

Examination of the emission spectrum of Si:ODA (Fig. S1B†), which reports the exciton energy distribution of Si QDs in the ensemble we have examined, suggests a second explanation for our system's reduced upconversion performance when excited at 730 nm. While Si QD emission spectra indicate the average exciton energy of the Si QDs in our ensemble is 1.6 eV, the exciton energy distribution of our ensemble is broad (0.29 meV FWHM). 730 nm photons have an energy of 1.70 eV that falls in the middle of the emission band of the Si QD ensemble, suggesting several QDs for which triplet energy transfer to 3EP would be energetically favored are not excited by the 730 nm laser source. We find restricting our kinetic model to only consider triplet transfer to 3EP from Si QDs with exciton energies of 1.70 eV or less skews the Si:3EP triplet equilibrium, placing only 20% of photoexcitations in the system on 3EP, thereby reducing the probability that any given collision between an excited Si:3EP QD and a *t*Bu_4_perylene molecule will result in successful triplet transfer.

This analysis suggests a natural way to skew this equilibrium towards surface-bound triplet transmitting molecules would be to either employ smaller Si QDs with larger exciton energies or replace perylene with a triplet transmitter with a lower triplet energy. Indeed, for the Si QD ensemble employed in our study our model predicts that replacement of perylene with tetracene (*E*_T_ = 1.25 eV)^62–64^ would significantly skew the equilibrium highlighted in Fig. 3C towards the organic transmitter, with >94% of the excited population residing on tetracene. We can likewise bias the triplet exciton population towards the organic transmitter by raising the energy of the Si QD donor. Our model predicts that a size-uniform sample of Si QDs with an exciton energy that is 215 meV larger than 3EP's triplet energy would yield an exciton distribution with >94% of its population resting on 3EP. Similar conclusions regarding the importance of introducing an energy gap between a QD triplet donor and molecular triplet acceptor has been noted by Rao and coworkers for a PbS:tetracene system^33^ and by other authors for small-molecule based triplet-fusion upconversion systems.^65,66^ We note that in our calculations, the upper limit for transfer of the exciton population from Si to the organic acceptor is ∼96% as we take the Si QDs to bind on average 3.2 acceptor molecules, the value that corresponds to the best fit to our Si:3EP transient absorption data. Assuming a Poisson distribution of these acceptors among Si QDs predicts ∼4% of the Si QD population binds no acceptor at all. This suggests combining energetic control of the Si QD:acceptor interface with a modest increase in the acceptor loading on Si QD surfaces can form an effective strategy towards achieving quantitative triplet energy transfer.

While these calculations are idealized as they ignore the influence of exciton decay pathways that compete with triplet exciton transfer, we note even in the presence of these decay channels, fast triplet extraction by a diffusing triplet acceptor such as *t*Bu_4_perylene can facilitate effective harvesting of energy from Si:3EP. Recent work examining tetracene-functionalized PbS QDs found that surface-bound tetracene molecules were able to transfer triplet excitons to rubrene energy acceptors in solution with a second-order rate constant of 28 μs^−1^ M^−1^.^67^ Assuming a similar second-order rate constant for triplet energy transfer from Si:3EP to *t*Bu_4_perylene yields a pseudo first-order rate constant of 1.4 × 10^−2^ μs^−1^ for the 0.5 mM *t*Bu_4_perylene concentration employed for measurements highlighted by Fig. 4. Addition of such a triplet harvesting pathway to our kinetic model suggests 35% of Si:3EP excitations can be readily harvested (Fig. S8†). This yield can be improved significantly, to 77%, *via* use of size-uniform Si QDs with an exciton energy 215 meV above that of 3EP's triplet energy. Eliminating trap states from these QDs and doubling their 3EP surface coverage would boost this yield further, to nearly 96%. These calculations highlight the importance of considering the equilibration of excited population when designing QD:molecule structures for photon conversion applications.

Our work also clearly demonstrates that triplet energy transfer from molecules to Si can readily occur on nanosecond timescales. This carries strong implications for the design of hybrid solar cells that pair singlet fission materials with Si in order to reduce thermalization losses in the Si layer.^18,22^ Theoretical calculations have shown that the highest gains in cell efficiency can be achieved if triplet excitons produced in the singlet fission material by high-energy photons are directly injected into Si *via* triplet energy transfer.^24^ Our work suggests covalent attachment of organic molecules to Si surfaces can ensure sufficient through-bond coupling to allow triplet transfer to occur from a molecular donor to Si. Moreover, the high density of states in Si *vs.* a molecule at its surface should favor this energy transfer. While there is certainly much work to be done to demonstrate the feasibility of this approach for bulk *vs.* nanocrystalline Si, our work indicates there are no issues intrinsic to Si:organic junctions that should limit this approach.

## Conclusions

In summary, we have shown that Si QDs can establish an excited state quasi equilibrium wherein triplet excitons dynamically shuttle between a QD and triplet-transmitting molecules bound to its surface. For the perylene-functionalized QDs we examine here, transient absorption experiments indicate this equilibrium is established on a 4.2 μs timescale and the population of triplet excitons residing in the perylene ligand shell is sufficiently high to enable creation of a photon upconversion system that operates with an efficiency of 1.5% under excitation fluences as low as 80 mW cm^−2^. Importantly, by altering the exciton energies of a Si QD and the transmitters bound to its surface, population can be skewed towards one side or the other of the Si:transmitter interface, suggesting a simple design criterion for creating Si:organic junctions that move energy in a desired direction for photon upconversion, singlet fission, or quantum information applications.

## Author contributions

T. H. and T. T. K. chemically functionalized Si QDs and performed all optical experiments. J. S. and L. M. prepared silicon quantum dots and contributed to data analysis and manuscript preparation. T. T.-T. T. synthesized vinyl-perylene ligands used to functionalize Si QDs. P. X. performed experiments on Si:9EA presented in the supporting information and described in the main text. K. W. obtained infrared spectra. S. T. R. devised the kinetic model used for analysis of triplet energy transfer rates and drafted the manuscript. L. M., M. L. T., and S. T. R. designed and oversaw the project.

## Conflicts of interest

The authors have no conflicts to declare.

## Supplementary Material

SC-012-D1SC00311A-s001
