# Supplementary material for: Bidirectional triplet exciton transfer between silicon nanocrystals and perylene
Source: Chem Sci. 2021 Apr 5;12(19):6737–46. doi: 10.1039/d1sc00311a (PMC8132999; doi:10.1039/d1sc00311a)
Supplement: SC-012-D1SC00311A-s001 [file SC-012-D1SC00311A-s001.pdf]

Electronic Supplementary Material (ESI) for Chemical Science.
This journal is © The Royal Society of Chemistry 2021

Supplementary Information

for

Bidirectional Triplet Exciton Transfer Between Silicon Nanocrystals and Perylene

Tingting Huang,[1] Timothy T. Koh,[1] Joseph Schwan,[2] Tiffany Tran,[1] Pan Xia,[3] Kefu Wang,[1] Lorenzo Mangolini,[2,3*] Ming L. Tang,[1,3*] and Sean T. Roberts[4,5*]

[1]*Department of Chemistry, University of California Riverside, Riverside, CA 92521, USA*
[2]*Department of Mechanical Engineering, University of California Riverside, Riverside, CA 92521, USA*
[3]*Materials Science & Engineering Program, University of California Riverside, Riverside, CA 92521, USA*
[4]*Department of Chemistry, The University of Texas at Austin, Austin, TX 78712, USA*
[5]*Center for Dynamics and Control of Materials, The University of Texas at Austin, Austin, TX 78712, USA*

[*] Authors to whom correspondence should be sent: lmangolini@engr.ucr.edu, mltang@ucr.edu, roberts@cm.utexas.edu,

1. Instrumentation

Absorption spectra were recorded on a Cary 5000 absorption spectrophotometer. Ensemble photoluminescence (e.g. fluorescence, photon upconversion, power dependence, etc.) spectra were recorded on a Maya 2000-Pro Spectrometer (Ocean Optics Inc.) with continuous wave (CW) solid-state lasers from Coherent Inc. (405 nm: OBIS LX 50 mW; 488 nm: OBIS LS 60 mW; 532 nm: Sapphire SF 532; 640 nm: OBIS LX 40 mW; 730 nm: OBIS LX 30 mW). Neutral density filters (Thorlabs) were used to tune the excitation source power without changing its beam size. Semrock notch filters were used to prevent excitation light from hitting the detector. Laser power was measured with a benchtop optical power and energy meter (2936R, Newport Corp.) and Si wand detector head (818-ST2/DB, Newport Corp.). Fourier Transform Infrared (FTIR) spectra were measured by a Thermo Fischer Scientific iS50 spectrometer using a diffuse reflectance (DRIFTS) tool.

X-ray diffraction (XRD) measurements were performed via a PANalytical Empyrean X-Ray system using Cu Kα radiation with a wavelength of 1.54 Å. TEM characterization of Si quantum dot (Si QD) samples was performed on a Tecnai T12 120 kV instrument. Samples were prepared by drop-casting Si QD solutions onto a supported thin-film carbon grid.

Nanosecond transient absorption (TA) measurements were performed using an enVISion spectrometer (Magnitude Instruments) employing 532 nm excitation pulses, with repetition rates varying from 200 Hz to 10 kHz depending on the scanned time delay range. The excitation energy density was ~300 $\mu J/cm^2$ for a 10 kHz repetition rate. Si QD samples were housed in a nitrogen glove box prior to TA measurements and placed into sealed cuvettes for TA experiments (Spectracell R-4001-T). Oxidation of NC samples was checked by measuring changes in their TA kinetics at a probe wavelength 505 nm, which denotes a spectral peak in the excited state absorption of 3-ethylperylene (3EP)'s lowest-excited triplet (T_1) state.

2. Sample Preparation and Characterization

A) Materials

1-octadecene (ODE, tech., 90%) was acquired from Acros Organics and was heated to 120 °C under vacuum to remove low boiling point impurities. Indocyanine green (95%) was purchased from Chem-Impex Int'l. Inc. Platinum octaethylporphyrin (PtOEP, 98%) used for triplet sensitization experiments was obtained from Sigma Aldrich. Dry acetone, dichloromethane, and toluene were purchased from Alfa Aesar and degassed with a JC Meyer solvent purification system. Dried methanol was acquired from Sigma Aldrich and was introduced into a glove box after degassing. Mesitylene (98%) was purchased from Sigma Aldrich and dried with molecular sieves. All other chemicals were used as received.

B) Organic synthesis

3-vinylperylene (3VP) was synthesized from 3-bromoperylene using Molander's organotrifluoroborate precursor, potassium vinyltrifluoroborate.[1] 2,5,8,11-tetra-*tert*-butylperylene, denoted in the main text and below as tBu_4perylene, was synthesized according to Askes et al.[2]

C) Silicon Quantum Dot Synthesis

Si QDs were produced using a capacitive non-thermal plasma, which allows for production of high-purity materials in the gas-phase. QDs are synthesized by inputting an argon-silane gas precursor mixture (1.36% SiH_4 in Argon) at a flow rate of 60 sccm into a 5 mm internal diameter borosilicate tube wherein it is exposed to a non-thermal plasma powered by a copper ring electrode on the outside of the tube. The electrode was connected to a radio frequency power supply (Advanced Energy RFPP RF-5S) outputting between 23 W and 30 W, which was coupled to the system via a matching network (MFJ-989D Versa Tuner). Particle size was controlled through material residence time in the plasma and gas pressure within the plasma region, which ranged between 1.32 Torr and 1.50 Torr for the samples used. Termination of Si QD surfaces with hydrogen atoms was accomplished through addition of a 100 sccm flow of hydrogen gas into the plasma afterglow. Following their synthesis and surface termination, QDs were collected on a

stainless steel mesh downstream of the reactor. Air-free transportation was done by closing valves both upstream and downstream of the collecting mesh, allowing for that section to be detached from the synthesis system and brought into a glovebox for processing.

D) Silicon Quantum Dot Radical-initiated Hydrosilylation

For the AIBN-initiated radical hydrosilylation of Si QDs, 0.425 mg of Si QDs were mixed with 1.0 mg of AIBN. Subsequently, 0.21 mol eq of ODE was added, corresponding to 0.06 mL of ODE. 3VP was added in different amounts to vary the mole ratio with respect to ODE, and the solution topped with toluene to a final volume of 1.12 mL. This solution was sealed, stirred, and heated in a nitrogen glovebox at 60 ºC for 15 hours. Successful QD hydrosilylation is signaled by the cloudy mixture turning clear.

Methanol was used to separate functionalized QDs from free 3VP precursor molecules. Each reaction mixture was divided into two 5 mL centrifuge tubes. The original vial was rinsed with 3 mL of methanol, resulting in a total volume of ~1.5 mL in each tube. Si:3EP was precipitated by centrifuging at 14,000 rpm for 20 mins. The precipitated Si:3EP in each centrifuge tube were individually redispersed in 0.5 mL of toluene, followed by addition of 1.5 mL of methanol and centrifugation at 14,200 rpm for an additional 20 min. This cleaning procedure was repeated three times and resulted in full removal of free 3VP and ODE molecules in solution as shown by the absence of 3VP features in absorption spectra of the supernatant.

E) Structural Characterization of Si:octadecane and Si:3EP QDs

XRD, TEM, and optical absorption and emission measurements were used to assess the crystallinity of Si QDs following radical-initiated hydrosilylation (**Figure S1A**). XRD measurements of octadecane-functionalized Si particles (Si:ODA) show peaks that match Si's diamond lattice. Scherrer analysis applied to the 111 peak at $2\theta = 28.6°$ yields an average crystalline domain size of 2.75 nm. TEM images of Si:ODA particles (**Figure S1A inset**) show the appearance of lattice fringes, although the low contrast of Si relative to the TEM background precludes an accurate analysis of the particle size distribution. Absorption spectra of Si:ODA particles (**Figure S1B**) show a broad, featureless emission extending throughout the visible region that is characteristic of Si QDs. Emission spectra of these particles show a broad peak centered at 780 nm that is consistent with an average QD diameter of 3.4 nm based on reported sizing curves.[3,4] This slight discrepancy in QD size predicted from emission spectra and the crystalline domain size indicated by XRD likely results from partial air exposure of samples used for XRD measurements, which can induce oxide growth that reduces the size of a QD's crystalline Si region.

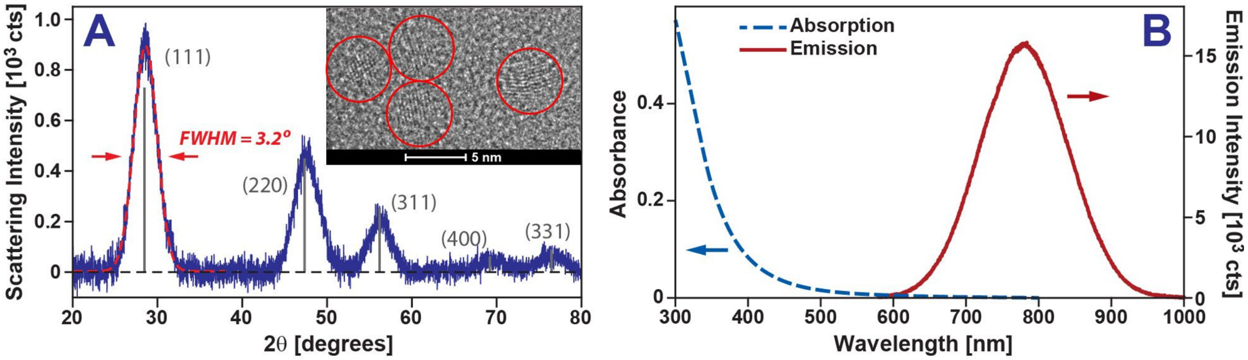

Figure S1: (A) XRD pattern of Si:ODA (blue) with the expected peak pattern for a Si diamond lattice (grey). (B) Absorption (blue) and emission spectra (red, $\lambda_{Ex} = 488$ nm) for Si:ODA.

Attachment of 3EP and ODA to hydrogen-terminated Si QDs was confirmed using FTIR spectroscopy performed using a DRIFTS stage (**Figure S2**). Following functionalization of Si particles with a mixed ligand shell containing 3EP and ODA, intense bands appear between 2800 – 3000 cm^{-1} and a weaker band at 1455 cm^{-1} that are characteristic of C-H stretching and bending modes of ODA, respectively. Weak features are also seen at 1590 cm^{-1}, ~1380 cm^{-1}, ~810 cm^{-1}, and 765 cm^{-1} that stem from 3EP. These bands

are reduced in amplitude relative to those of ODA due to the higher concentration of ODA of the surface. A small peak in the Si:3EP spectrum at 1017 cm^{-1} likely arises from Si-O stretching, indicating some surface oxidation of FTIR samples.

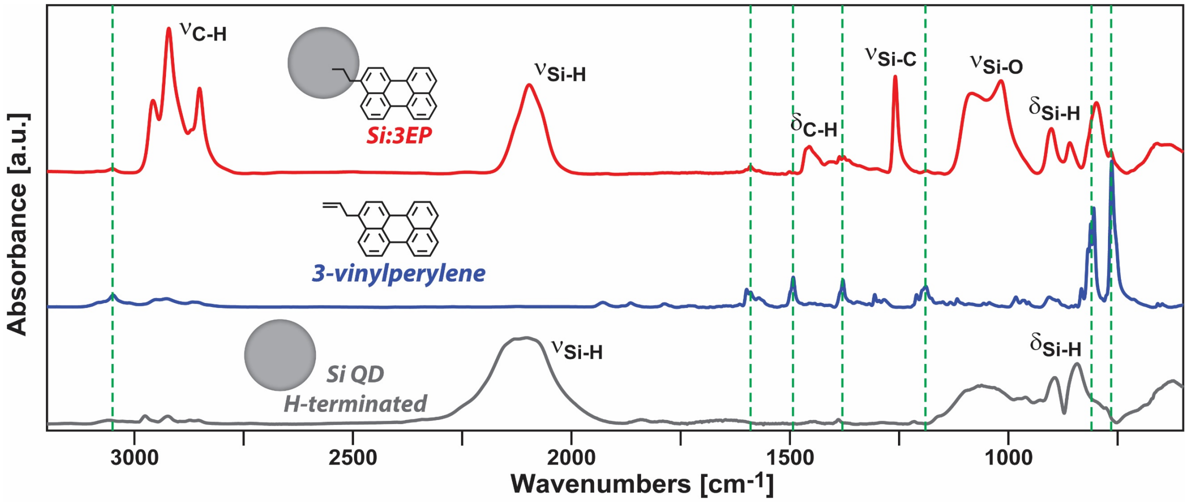

Figure S2: FTIR spectra of Si:3EP, 3VP, and hydrogen-terminated Si QDs. Bands appearing at 2700 – 3000 cm^{-1} and ~1450 cm^{-1} confirm attachment of ODA to Si while bands at ~1600 cm^{-1}, ~1375 cm^{-1}, ~815 cm^{-1}, and ~765 cm^{-1} indicate attachment of 3EP, albeit in lower concentration.

F) Attempted Functionalization of Si QDs with 3EP via Thermal Hydrosilylation

The radical-initiated hydrosilylation approach described in section C was used to attach 3VP to Si QDs as attempts to use a thermal hydrosilylation method previously employed to functionalize hydrogen-terminated Si QDs with 9-ethylanthracene[5] yielded a black precipitate that showed no indication of photon upconversion when mixed with perylene-based triplet fusion annihilator. Rather this precipitate exhibited a broad, featureless emission spectrum, when excited at 532 nm (**Figure S3**) suggesting it consists of an inhomogeneous mixture of nano-silicon and molecular species. This is perhaps not surprising as vinyl groups, such as that contained by 3VP and ODE, are known to polymerize when heated to the temperature used for thermal hydrosilylation (180 °C).[6] Use of low-temperature, radical-initiated hydrosilylation instead yields a dispersible functionalized Si material that readily undergoes photon upconversion when mixed with perylene-based triplet fusion annihilators.

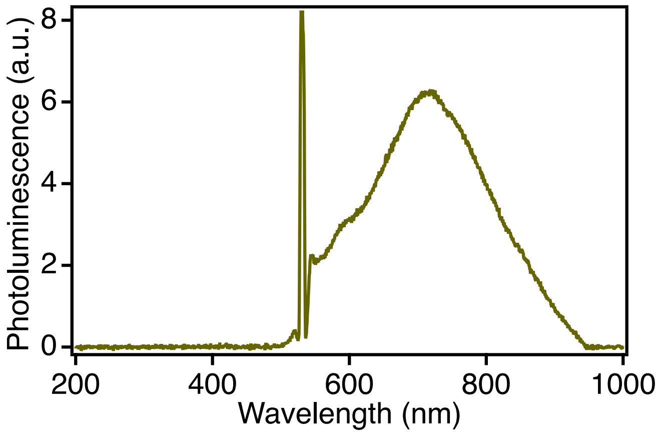

Figure S3: Thermal hydrosilylation of hydrogen-terminated Si QDs with 3VP:ODE = 0.02:1 at 180 ºC produced a black precipitate that exhibited the plotted spectrally-broad emission spectrum when excited at 532 nm in toluene. This precipitate produced no photon upconversion when mixed with perylene-based triplet fusion annihilators.

3. Estimating the average number of surface-bound 3EP ligands, $\langle N_{3EP} \rangle$

We used absorption spectra of solutions of functionalized Si:3EP and Si:ODA to estimate the average number of 3EP molecules that attach to Si QDs, $\langle N_{3EP} \rangle$, within different Si:3EP samples. To assess Si QD concentration, we examine the extinction of solutions at a wavelength of 488 nm as Si QDs show an appreciable absorption at this wavelength while 3EP does not. Measurements of the extinction coefficient of Si QDs at 488 nm span a range of reported values that we estimate to fall between 1.3×10^4 $M^{-1}cm^{-1}$ to 7.0×10^4 $M^{-1}cm^{-1}$.[3,4,7] We note that some of the reported estimates for the Si QD extinction we have employed correspond to values measured for QDs that differ in size from the 3.4 nm diameter QDs we employ. We have scaled these values assuming the QD extinction scales linearly with their volume. To calculate the concentration of 3EP molecules in a sample, we assume the extinction coefficient of 3EP when attached to Si in toluene at its absorption maximum is unchanged from that of perylene in cyclohexane (39000 $M^{-1}cm^{-1}$ at 436 nm).[8] While electronic coupling between Si and 3EP may question the accuracy of this assumption, small variations in the perylene extinction coefficient will have no impact on our qualitative conclusion that equilibration of the excited triplet exciton population between a Si QD and 3EP molecules bound to its surface is needed to reproduce our reported TA spectra. Using the extinction values above, we estimate between 0.9 – 4.6 3EP molecules are on average bound to the Si QDs we employed for transient absorption measurements in the main text (**Figures 2** and **3**) whereas 1.2 – 6.5 3EP molecules on average were bound to Si QDs used for photon upconversion measurements (**Figure 4**).

4. Triplet Sensitization of 3-vinylperylene

Following photoexcitation of Si, TA spectra of Si:3EP QDs show growth of a sharp photoinduced absorption peaked at 505 nm and a similarly sharp photobleach at 450 nm. These features are similar to bands previously assigned to T_1 state of perylene,[9] suggesting their appearance signals triplet energy transfer from excited Si QDs to 3EP. To test this assignment, we measured the TA spectrum of 3-vinylperylene (3VP) in toluene via a triplet sensitization experiment employing platinum octaethylporphyrin (PtOEP) as a sensitizer. A mixture of 3VP and PtOEP in toluene was prepared by mixing 0.05 mL of a 1.4 mg/mL PtOEP solution with 0.35 mL of a 1 mg/mL 3VP solution and 4.6 mL of toluene. This yields a mixture containing 0.35 mg/mL of 3VP and 0.07 mg/mL of PtOEP, which makes the mass ratio of PtOEP to 3VP 20%. Photoexcitation of PtOEP's Q-band at 532 nm places it into its lowest excited singlet state, which rapidly intersystem crosses to its triplet state on a ~165 fs timescale.[10] As PtOEP possess a triplet energy of 1.9 eV[11] that is higher than that of 3VP (~1.53 eV), diffusional collisions of photoexcited PtOEP with ground state 3VP molecules can result in triplet energy transfer from PtOEP to 3VP.

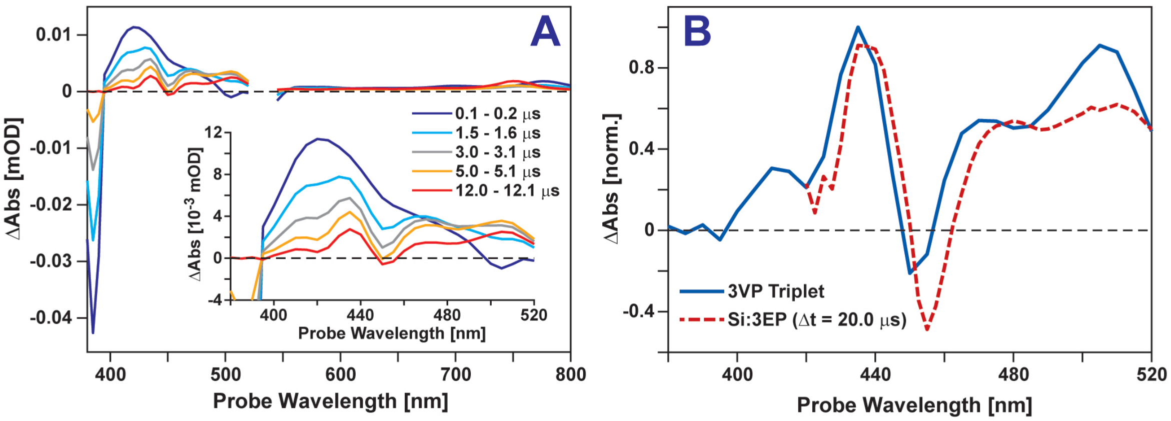

Figure S4: (A) TA spectra of a PtOEP:3VP mixture following PtOEP excitation at 532 nm. As spectral signatures from PtOEP's T_1 state decay, new features appear between 400 – 520 nm that are due to 3VP's T_1 state. (B) Comparison of the TA spectrum of 3VP's T_1 state to that seen at long time delays (20 μs) following photoexcitation of Si:3EP signal triplet energy transfer from Si QDs to 3EP molecules at their surface.

Figure S4A plots TA spectra of the PtOEP:3VP solution following PtOEP photoexcitation at 532 nm. At short time delays (100 – 200 ns), a prominent photobleaching transition is seen at 385 nm from PtOEP's Soret band. At longer wavelengths, a broad photoinduced absorption peaked at 420 nm appears that matches well prior assignments of PtOEP's T_1 state.[10] Over time, these features from PtOEP's T_1 state decay, leaving behind a series of peaks at 435, 472, and 505 nm as well as a negative feature at 452 nm. This latter feature agrees well with the primary absorption band of 3VP's ground state while the induced absorption bands agree with prior assignments of perylene's T_1 state,[9] leading us to assign these features to triplet energy transfer from PtOEP to 3VP.

Importantly, TA spectra of Si:3EP following the decay of Si QD photoinduced absorption reveal features that strongly resemble 3VP's T_1 TA spectrum but shifted to slightly longer wavelengths (**Figure S4B**). Such a shift is consistent with that seen for ground state absorption spectra of 3VP upon binding to Si (**Figure 1, main text**) and has previously been noted for similar molecules, such as 9-ethylanthracene,[5] upon Si attachment. This leads us to conclude that the appearance of these features in TA spectra of Si:3EP signal triplet exciton transfer from Si to 3EP.

5. Exciton Dynamics of Si:ODA

Figure 2A of the main text shows photoexcitation of Si:ODA particles produces a photoinduced absorption band that extends across the visible spectrum. This band has previously been assigned to intraband transitions of photoexcited carriers[5,12–15] and its relaxation is non-exponential, which has been attributed to the presence of carrier traps dispersed among a Si QD ensemble. Previously, we showed the nonexponential relaxation kinetics of Si QD ensembles could be well reproduced by a kinetic model that assumed different types of traps with characteristic carrier trapping timescales were distributed among the ensemble.[5] Briefly, this model takes the relaxation rate of a QD within the ensemble to be given by:

$$k_{Si} = k_{Si}^{0} + \sum_{m} N_m k_m \quad \text{(S1)}$$

Here, k_{Si}^0 represents the intrinsic relaxation rate of a Si QD in the absence of carrier trapping while the sum runs over different types of characteristic traps, $\{m\}$. Each type of trap is taken to have a characteristic trapping rate, k_m, while N_m represents the number of traps of a specific type on a given QD. Assuming these traps are distributed among QDs according to a Poisson distribution, the excited Si QD exciton population as a function of time can be solved for analytically:[16]

$$\left[Si^*(t)\right] = \exp\left(-k_{Si}^{0}t + \sum_{m}\langle N_m\rangle\left(\exp\left(-k_m t\right)-1\right)\right) \quad \text{(S2)}$$

Here, $\langle N_m\rangle$ represents the average number of each type of trap contained by Si QDs within the ensemble. Consistent with our prior work examining Si QDs grown via nonthermal plasma synthesis,[5] we find two types of traps with distinct relaxation rates are needed to model our spectra (**Figure S5**).

Interestingly, comparing the relaxation rates we obtain for the set of Si QDs employed in our present study, which were functionalized with ODA via a radical-initiated hydrosilylation method, against the relaxation rates for Si QDs functionalized with ODA via thermal hydrosilylation[5] reveals that QDs employed in the present study exhibit a longer exciton lifetime due to a reduction in trap density (**Table S1**). We leave to future work an assessment of the extent to which this reduced trap density reflects the different methods used to functionalize these samples, improvements to our handling of Si QDs post-synthesis to reduce surface oxide growth, or the larger size of the QDs employed in the present study. With the fit parameters from **Table S1** in hand however, we can use them to model the relaxation behavior of excitons within 3VP-functionalized Si QDs, as outlined in section 6.

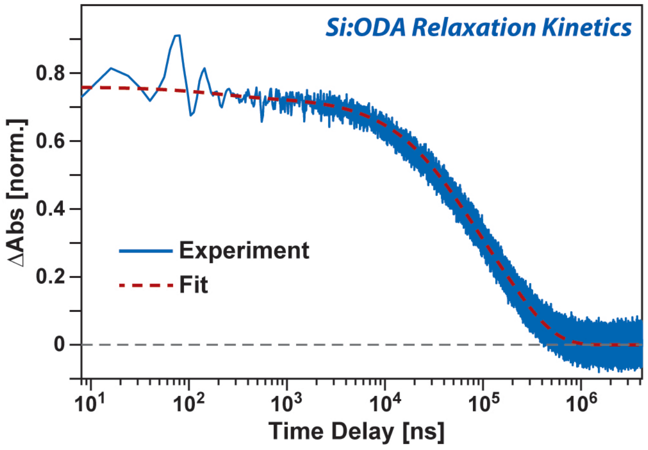

Figure S5: TA kinetics of Si:ODA dissolved in toluene measured at a probe wavelength of 1000 nm following 532 nm photoexcitation. This trace reports the relaxation rate of photogenerated excitons within ODA-terminated Si QDs and can be well reproduced by Eq. S2 (red dashed).

	$1/k_{Si}^{0}$	$\langle N_1 \rangle$	$1/k_1$	$\langle N_2 \rangle$	$1/k_2$	$\langle N_3 \rangle$	$1/k_3$
Current Study	210 μs	0.040	197 ns	0.46	57.4 μs	--	--
Reference [5]	180 μs	0.51	4.95 ns	0.43	213 ns	0.47	5.59 μs

Table S1: Best-fit parameters for the relaxation kinetics of Si:ODA extracted from data in **Figure S5** vs. those reported in Ref. 5. Only two types of exciton traps were needed to model the behavior of Si:ODA reported here whereas three traps were required to model data reported in Ref. 5.

6. Kinetic Model for Si-to-3EP Triplet Exciton Transfer

Figure 3B of the main text displays a Jablonski plot that summarizes the kinetic model we use to model Si-to-3EP triplet exciton transfer. This model is similar to one we previously employed to describe triplet exciton transfer from Si QDs to 9-ethylanthracene,[5] but now explicitly allows for back energy transfer from the triplet acceptor to the Si QD to which they bind. Briefly, this model assumes our samples are comprised of an ensemble of QDs with different exciton energies that reflect their size distribution. Individual QDs within this ensemble can bind differing numbers of 3EP molecules. Following photoexcitation, a Si QD can either transfer a triplet exciton to one of the 3EP molecules on its surface or relax to its ground state. This behavior is described by the following differential equation:

$$\frac{d[Si^*]}{dt} = -k_{Si}[Si^*] - k_{TET}\left(E_a^{TET}\right)N_{3EP}[Si^*] + k_{TEBT}\left(E_a^{TEBT}\right)[3EP^*] \tag{S3a}$$

Here, $[Si^*]$ and $[3EP^*]$ represent the concentration of Si QD excitons and 3EP spin-triplet excitons, k_{Si} denotes the deactivation rate of Si QD excitons, k_{TET} and k_{TEBT} respectively signify the rates of triplet energy transfer from Si to 3EP and from 3EP to Si, and N_{3EP} specifies the number of 3EP molecules bound to a QD's surface. Carrier traps on a Si QD are assumed to facilitate exciton recombination, hence we assume rates for carrier trapping add to k_{Si} according to Eq. S1. Similarly, we describe the population of 3EP triplet excitons using:

$$\frac{d[3EP^*]}{dt} = -k_{3EP}[3EP^*] + k_{TET}\left(E_a^{TET}\right)N_{3EP}[Si^*] - k_{TEBT}\left(E_a^{TEBT}\right)[3EP^*] \tag{S3b}$$

Here, k_{3EP} denotes the rate of decay of 3EP triplet excitons to the ground state. To account for differences in the triplet exciton energy of a Si QD, E_{Si}, and a 3EP molecule bound to its surface, E_{3EP}, we use a Miller-

Abrahams rate expression[17,18] to describe k_{TET}:

$$k_{TET}\left(E_a^{TET}\right)=\begin{cases} k_{TET}^0 \exp\left(-E_a^{TET}/k_bT\right) & \text{if } E_a^{TET}>0 \\ k_{TET}^0 & \text{if } E_a^{TET}\le 0 \end{cases} \quad \text{where} \quad E_a^{TET}=E_{3EP}-E_{Si} \tag{S4a}$$

An analogous Miller-Abrahams expression can be written for the rate of triplet energy back transfer from a 3EP molecule to the QD to which it is bound:

$$k_{TEBT}\left(E_a^{TEBT}\right)=\begin{cases} k_{TEBT}^0 \exp\left(-E_a^{TEBT}/k_bT\right) & \text{if } E_a^{TEBT}>0 \\ k_{TEBT}^0 & \text{if } E_a^{TEBT}\le 0 \end{cases} \quad \text{where} \quad E_a^{TEBT}=E_{Si}-E_{3EP} \tag{S4b}$$

As Eq. S4a and S4b are a pair of linear, coupled first order differential equations, they can be solved analytically under the initial condition that the excitation pulse only excites the Si QDs within a sample:

$$\left[Si^*(t)\right]=\frac{\left[Si^*\right]_0}{2\Omega}\left(\left(\Omega-\Delta\right)e^{\lambda_+t}+\left(\Omega+\Delta\right)e^{\lambda_-t}\right) \tag{S5a}$$

$$\left[3EP^*(t)\right]=\frac{\left[Si^*\right]_0 N_{3EP}k_{TET}}{2\Omega}\left(e^{\lambda_+t}-e^{\lambda_-t}\right) \tag{S5b}$$

where:

$$\lambda_\pm=-\frac{\left(k_{Si}+N_{3EP}k_{TET}+k_{3EP}+k_{TEBT}\right)}{2}\pm\Omega,\quad \Delta=\frac{k_{Si}+N_{3EP}k_{TET}-k_{3EP}-k_{TEBT}}{2},\quad \Omega=\sqrt{\Delta^2+N_{3EP}k_{TET}k_{TEBT}},$$

and $[Si^*]_0$ denotes the concentration of Si QD excitons excited by the laser pulse at $t = 0$.

To account for the spread of QD exciton energies, the distribution of carrier traps among QDs, and heterogeneity in the number of 3EP molecules bound to individual QDs within the ensemble, we compute exciton populations via Eq. S5a and S5b using a series of values for the Si QD exciton energy (E_{Si}), the number of traps of each type on a QD ($\{N_m\}$, Eq. S1), and the number of 3EP molecules bound to a QD, N_{3EP}. We then construct the dynamics of the ensemble by taking a weighted average of the populations calculated for different pairings of these parameters wherein the weights are determined by assuming the distribution of exciton energies follows the emission spectrum of Si:ODA and the number traps within Si QDs and 3EP molecules bound to their surface are distributed according to Poisson statistics.

While at first glance it may seem as if this model has several free parameters, many of them are constrained by independent measurements. For example, the average number of 3EP molecules bound to Si QDs within an ensemble, $\langle N_{3EP}\rangle$ is determined from their ground state absorption spectrum. Likewise, parameters governing the intrinsic and trap-mediated relaxation of Si QDs to their ground state are constrained by TA measurements of Si:ODA.

Table S2 summarizes best fit parameters used to reproduce TA spectra showing the growth and decay of 3EP triplet excitons following photoexcitation of Si:3EP. As seen in **Figure 3A** of the main text, the fit reproduces well the dynamics of the 3EP triplet population over 6 decades of time with only a few adjustable parameters. To fit this population fully, we found we needed to slightly alter the average number of carrier traps distributed throughout the Si:3EP population relative to those found when fitting TA spectra of Si:ODA (**Table S1**). Such changes to our model were also found necessary in prior work examining energy transfer from Si QDs to surface-bound 9-ethylanthracene molecules[5] and could reflect slight changes in the surface structure of Si QDs upon functionalization with triplet exciton acceptors.

	Parameter	*Method of Determination*
$\langle N_{3EP} \rangle$	3.2	Range estimated from absorption spectra of Si:3EP
$1/k_{TET}^{0}$	4.22 μs	Floated to fit Si:3EP dynamics
$1/k_{TEBT}^{0}$	22.0 ns	Floated to fit Si:3EP dynamics
$1/k_{3EP}$	2.26 ms	Floated to fit Si:3EP dynamics
$1/k_{Si}^{0}$	210 μs	Constrained when fitting Si:3EP based on Si:ODA decay
$\langle N_1 \rangle$	0.17	Floated to fit Si:3EP dynamics
$1/k_1$	197 ns	Constrained when fitting Si:3EP based on Si:ODA decay
$\langle N_2 \rangle$	0.58	Floated to fit Si:3EP dynamics
$1/k_2$	57.4 μs	Constrained when fitting Si:3EP based on Si:ODA decay
E_{3EP}	1.53 eV	Estimated from References 19–23

Table S2: Best-fit parameters for the kinetic model described by Eq. S5a and S5b used to describe triplet energy transfer between Si QDs and surface-bound 3EP molecules.

7. Transient Absorption Spectra of 9-Ethylanthracene Functionalized Si QDs

A key finding of our report is that we observe equilibration of an excited spin-triplet exciton population distributed between Si QDs and 3EP molecules covalently tethered to their surface. This equilibration is enabled by the long lifetime of these triplet excitons relative to timescales for their exchange between Si and 3EP. We note this finding lies in contrast to results we recently reported on a related system comprised of Si QDs functionalized with 9-ethylanthracene (9EA) ligands.[5] Upon photoexcitation of Si in this latter system, we found triplet energy transfer to occur from Si to 9EA with a time constant of 15.2 ns followed by decay of the 9EA triplet state back to the ground state on a 1.16 μs timescale. This decay timescale is accelerated relative to the triplet lifetime of 9EA molecules freely diffusing in deoxygenated solution (~3 ms),[24] suggesting involvement of additional triplet quenching pathways in our prior measurements. In the time since this work, we have improved our sample preparation and handling conditions to allow for better removal of oxygen and reduced formation of Si QD defects that can quench triplet excitons, as discussed in section 5. As such, we felt it prudent to record the photoexcited dynamics of Si:9EA samples prepared with these improved sample protocols in place.

Figure S6 shows TA spectra of Si:9EA dissolved in toluene. To prepare these samples, hydrogen-terminated Si QDs were synthesized in accordance with methods detailed in section 2C followed by functionalization with a mixed ligand shell containing a mixture of 9EA and ODA using thermal hydrosilylation as detailed in Ref. 5. Following photoexcitation of Si, we observe growth of an induced absorption band peaked at ~435 nm over the course of ~10 ns that indicates population of 9EA's T_1 state, consistent with our prior work.[5] However, rather than exhibiting the 1.16 μs lifetime we previously reported for this band, we find the 9EA T_1 state persists for several tens of microseconds. By the longest time delay we report (58 μs), this band has only decayed from its maximum intensity by ~35%, suggesting a T_1 state lifetime of at least 135 μs. Interestingly, we find the rate of decay of the 9EA T_1 state appears to match that of induced absorption bands stemming from intraband transitions of excitons within the Si QDs seen at longer wavelengths. This matching of the decay rate between the excited 9EA and Si QD population suggests establishment of a dynamic equilibrium that allows excitons to move between these materials on a timescale that is fast relative to the rate of triplet exciton decay, which is consistent with the observations we report for Si:3EP in the main text.

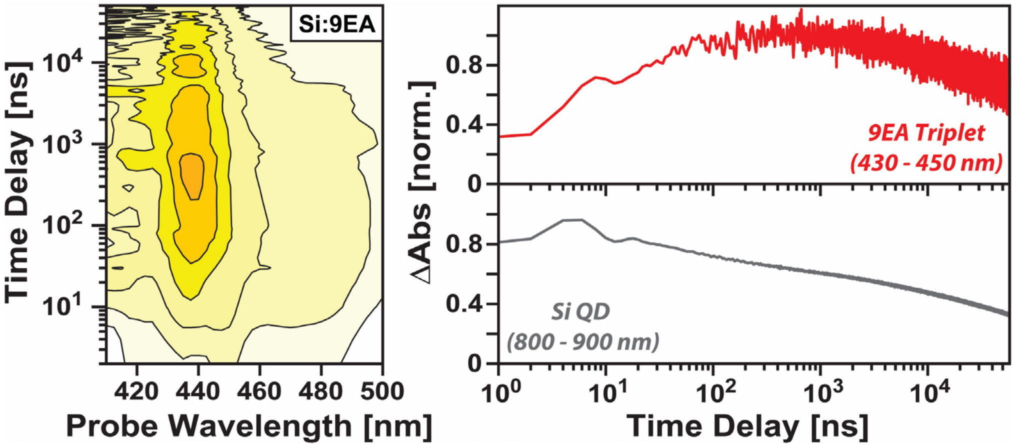

Figure S6: (Left) TA spectra of Si:9EA dissolved in toluene following photoexcitation of Si at 532 nm. Growth of a photoinduced absorption peaked at 435 nm is attributed to formation of the 9EA T_1 state. (Right) Growth and decay kinetics of the 9EA T_1 state (red) and Si QD exciton induced absorption (grey). Both bands decay with similar rates over several tens of microseconds, suggesting equilibration of triplet exciton population between 9EA and Si QD states.

8. Photon Upconversion with Si:3EP as a Triplet Photosensitizer

Samples for photon upconversion measurements were prepared by dispersing the contents of a centrifuge tube discussed in section 2D in a toluene solution containing 0.5 mM tBu_4perylene. Solutions were transferred to fluorescence cuvettes under air-free conditions in a glovebox (Starna 3-Q-10-GL14-S). The optical density at 532 nm of the Si QDs in this solution was held in the range of 0.1 to 0.2 for accurate measurements.

Our group has previously reported on the calculation of upconversion quantum yields (Φ_{UC}).[25] We employ Rhodamine 6G (R6G, 95% fluorescence quantum yield) as an emission intensity reference. The expression used to compute Φ_{UC} from emission spectra of Si:3EP in the presence of triplet fusion annihilators is given by Equation S6.

$$\begin{aligned}\Phi_{UC} &= 2*\Phi_{reference}*\frac{photons\ aborbed\ by\ reference}{photons\ absorbed\ by\ upconversion}*\frac{upconversion\ PL}{reference\ PL} \\ &= 2*\Phi_{R6G}*\frac{n^2_{Upconversion}}{n^2_{R6G}}*\frac{[Area]_{Upconversion}}{[Area]_{R6G}}*\frac{1-10^{-OD_{R6G}}}{1-10^{-OD_{Si\ QD}}}\end{aligned} \quad (S6)$$

where Φ_{RG6} is the fluorescence quantum yield of R6G, $n_{Upconversion}$ and n_{RG6} denote the refractive indices of the solvents for Si QD upconversion (toluene) and R6G reference (ethanol) solutions, $[Area]_{Upconversion}$ and $[Area]_{R6G}$ are the integrated areas of the photon upconversion and R6G emission peaks, and $OD_{Si\ QD}$ and OD_{R6G} denote the absorbance of Si QDs and R6G at the excitation wavelength used to excite each sample.

9. Estimating the Efficiency of Triplet Exciton Extraction from Si:3EP

While our kinetic model indicates photoexcitation of Si:3EP creates a quasi-equilibrium distribution with some excitations centered on Si QDs and others localized on 3EP molecules, the dynamic nature of this equilibrium suggests many of these excitations can be harvested by triplet exciton acceptors diffusing in solution. To examine the efficiency with which triplet excitons can be extracted from Si:3EP, we have added to our kinetic model an additional energy transfer pathway that allows for the transfer of triplet excitons from surface-bound 3EP molecules to an acceptor in solution, such as tBu_4perylene (**Figure S7**). Recent work that examined triplet exciton extraction from tetracene-functionalized PbS QDs found triplet

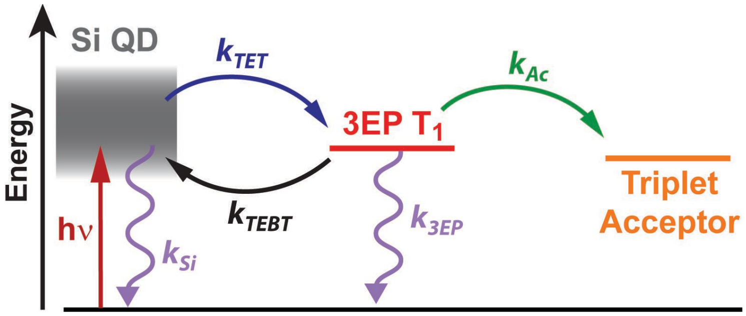

Figure S7: Kinetic model used to estimate the fraction of triplet excitons that can be extracted from Si:3EP.

harvesting by rubrene triplet acceptors occurs with a second-order rate constant of 28 $\mu s^{-1}M^{-1}$.[26] Assuming a similar rate holds for extraction of triplets from Si:3EP yields a pseudo first-order rate constant for triplet extraction of 1.4×10^{-2} μs^{-1} for an acceptor concentration matching that used for photon upconversion measurements reported in the main text (**Figure 4**). Inserting a similar first-order rate constant to our model leads to the following modifications to Eq. S3a and S3b:

$$\frac{d[Si^*]}{dt} = -k_{Si}[Si^*] - k_{TET}\left(E_a^{TET}\right)N_{3EP}[Si^*] + k_{TEBT}\left(E_a^{TEBT}\right)[3EP^*] \quad \text{(S7a)}$$

$$\frac{d[3EP^*]}{dt} = -\left(k_{3EP} + k_{Ac}\right)[3EP^*] + k_{TET}\left(E_a^{TET}\right)N_{3EP}[Si^*] - k_{TEBT}\left(E_a^{TEBT}\right)[3EP^*] \quad \text{(S7b)}$$

$$\frac{d[Ac^*]}{dt} = k_{Ac}[3EP^*] \quad \text{(S7c)}$$

where $[Ac^*]$ is the concentration of excited triplet acceptors and k_{Ac} is the pseudo first-order rate constant describing triplet energy transfer from 3EP to these acceptors. This set of linearly-coupled differential equations can be solved to yield the following values for the Si QD exciton population ($[Si^*]$), 3EP triplet exciton population ($[3EP^*]$), and $[Ac^*]$:

$$\left[Si^*(t)\right] = \frac{[Si^*]_0}{2\Omega}\left((\Omega - \Delta)e^{\lambda_+ t} + (\Omega + \Delta)e^{\lambda_- t}\right) \quad \text{(S8a)}$$

$$\left[3EP^*(t)\right] = \frac{[Si^*]_0 N_{3EP} k_{TET}}{2\Omega}\left(e^{\lambda_+ t} - e^{\lambda_- t}\right) \quad \text{(S8b)}$$

$$\left[Ac^*(t)\right] = \frac{[Si^*]_0 N_{3EP} k_{TET} k_{Ac}}{2\Omega}\left(\frac{1}{\lambda_+}\left(e^{\lambda_+ t} - 1\right) - \frac{1}{\lambda_-}\left(e^{\lambda_- t} - 1\right)\right) \quad \text{(S8c)}$$

where the values of Ω, Δ, and $\lambda_\pm$ have been modified according to:

$$\lambda_\pm = -\frac{\left(k_{Si} + N_{3EP}k_{TET} + k_{3EP} + k_{Ac} + k_{TEBT}\right)}{2} \pm \Omega, \quad \Delta = \frac{k_{Si} + N_{3EP}k_{TET} - k_{3EP} - k_{Ac} - k_{TEBT}}{2}, \text{ and}$$

$$\Omega = \sqrt{\Delta^2 + N_{3EP}k_{TET}k_{TEBT}} .$$

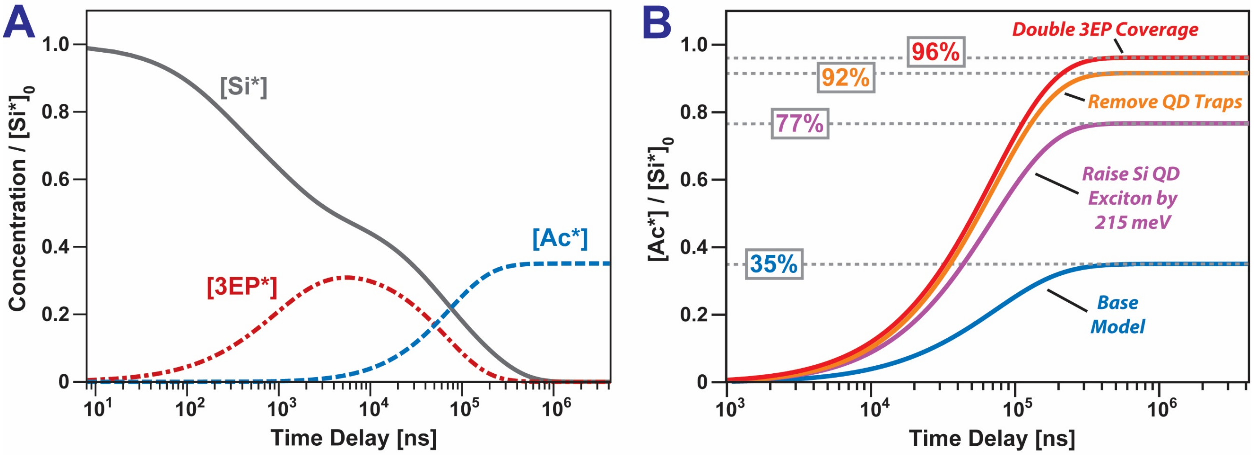

Figure S8: (A) Concentration of photoexcited species computed using the kinetic model illustrated by **Figure S7** and Eq. S7. (B) Taking k_{Ac} to be equal to 1.4 × 10^{-2} μs^{-1}, we predict 35% of excitons produced by the Si:3EP system described in the main text can be extracted (blue). Our model predicts this yield can be further increased to 77% if the Si QD exciton energy is raised by 215 meV (purple), 92% if the exciton energy is raised together with removing Si QD exciton traps (gold), and to 96% if $\langle N_{3EP} \rangle$ is doubled together with raising the exciton energy and eliminating traps (red).

Figure S8A plots the population dyanamics computed for this model using our experimentally determined parameters for Si:3EP (**Table S2**) together with a value of k_{Ac} of 1.4 × 10^{-2} μs^{-1}. We find that the dynamic equilibrium extablished between Si and 3EP allows for over a third of these excitations, 35%, to be extracted by the acceptor.

To identify steps that can be taken to improve this yield, we have systematically examined the role of different exciton loss pathways in our model (**Figure S8B**). We find the largest gains in triplet extraction efficiency can be achieved by shifting the exciton equilibrium in favor of 3EP by creating a moderate energy difference between the Si QD and 3EP exciton states. Specifically, by narrowing the size dispersion of the Si QDs and raising their exciton energy such that it lies 215 meV above the triplet energy of 3EP (1.53 eV), or model predicts the triplet extraction yield can be improved to 77% (**Figure S8B, purple**). Improving the processing of Si QDs to eliminate traps that quench excitons (**Table 1**) can further push the extraction yield to 92% (**Figure S8B, gold**). This value can be boosted to nearly 96% if additional steps are taken to ensure nearly all Si QDs in the ensemble bind a 3EP molecule to their surface by doubling $\langle N_{3EP} \rangle$ (**Figure S8B, red**). We note that speeding k_{Ac} will likewise act to improve each of these predicted yields.

10. References

(1) Molander, G. A.; Brown, A. R. Suzuki−Miyaura Cross-Coupling Reactions of Potassium Vinyltrifluoroborate with Aryl and Heteroaryl Electrophiles. *J. Org. Chem.* **2006**, *71* (26), 9681–9686.

(2) Askes, S. H. C.; Pomp, W.; Hopkins, S. L.; Kros, A.; Wu, S.; Schmidt, T.; Bonnet, S. Imaging Upconverting Polymersomes in Cancer Cells: Biocompatible Antioxidants Brighten Triplet-Triplet Annihilation Upconversion. *Small* **2016**, *12* (40), 5579–5590.

(3) Carroll, G. M.; Limpens, R.; Neale, N. R. Tuning Confinement in Colloidal Silicon Nanocrystals with Saturated Surface Ligands. *Nano Lett.* **2018**, *18* (5), 3118–3124.

(4) Wheeler, L. M.; Anderson, N. C.; Palomaki, P. K. B.; Blackburn, J. L.; Johnson, J. C.; Neale, N. R. Silyl Radical Abstraction in the Functionalization of Plasma-Synthesized Silicon Nanocrystals. *Chem. Mater.* **2015**, *27* (19), 6869–6878.

(5) Xia, P.; Raulerson, E. K.; Coleman, D.; Gerke, C. S.; Mangolini, L.; Tang, M. L.; Roberts, S. T. Achieving Spin-Triplet Exciton Transfer between Silicon and Molecular Acceptors for Photon Upconversion. *Nat. Chem.* **2020**, *12* (2), 137–144.

(6) Dhaene, E.; Billet, J.; Bennett, E.; Van Driessche, I.; De Roo, J. The Trouble with ODE: Polymerization during Nanocrystal Synthesis. *Nano Lett.* **2019**, *19* (10), 7411–7417.

(7) Lee, B. G.; Luo, J.-W.; Neale, N. R.; Beard, M. C.; Hiller, D.; Zacharias, M.; Stradins, P.; Zunger, A. Quasi-Direct Optical Transitions in Silicon Nanocrystals with Intensity Exceeding the Bulk. *Nano Lett.* **2016**, *16* (3), 1583–1589.

(8) Berlman, I. B. *Handbook of Fluoresence Spectra of Aromatic Molecules*, 2nd ed.; Academic Press: New York, 1971.

(9) Ye, C.; Gray, V.; Mårtensson, J.; Börjesson, K. Annihilation Versus Excimer Formation by the Triplet Pair in Triplet–Triplet Annihilation Photon Upconversion. *J. Am. Chem. Soc.* **2019**, *141* (24), 9578–9584.

(10) Roberts, S. T.; Schlenker, C. W.; Barlier, V.; McAnally, R. E.; Zhang, Y.; Mastron, J. N.; Thompson, M. E.; Bradforth, S. E. Observation of Triplet Exciton Formation in a Platinum-Sensitized Organic Photovoltaic Device. *J. Phys. Chem. Lett.* **2011**, *2*, 48–54.

(11) Baldo, M. A.; O'Brien, D. F.; You, Y.; Shoustikov, A.; Sibley, S.; Thompson, M. E.; Forrest, S. R. Highly Efficient Phosphorescent Emission from Organic Electroluminescent Devices. *Nature* **1998**, *395*, 151–154.

(12) Beard, M. C.; Knutsen, K. P.; Yu, P.; Luther, J. M.; Song, Q.; Metzger, W. K.; Ellingson, R. J.; Nozik, A. J. Multiple Exciton Generation in Colloidal Silicon Nanocrystals. *Nano Lett.* **2007**, *7* (8), 2506–2512.

(13) Limpens, R.; Pach, G. F.; Neale, N. R. Nonthermal Plasma-Synthesized Phosphorus–Boron Co-Doped Si Nanocrystals: A New Approach to Nontoxic NIR-Emitters. *Chem. Mater.* **2019**, *31* (12), 4426–4435.

(14) Trinh, M. T.; Limpens, R.; Gregorkiewicz, T. Experimental Investigations and Modeling of Auger Recombination in Silicon Nanocrystals. *J. Phys. Chem. C* **2013**, *117* (11), 5963–5968.

(15) Stolle, C. J.; Lu, X.; Yu, Y.; Schaller, R. D.; Korgel, B. A. Efficient Carrier Multiplication in Colloidal Silicon Nanorods. *Nano Lett.* **2017**, *17* (9), 5580–5586.

(16) Utterback, J. K.; Wilker, M. B.; Brown, K. A.; King, P. W.; Eaves, J. D.; Dukovic, G. Competition between Electron Transfer, Trapping, and Recombination in CdS Nanorod–Hydrogenase Complexes. *Phys. Chem. Chem. Phys.* **2015**, *17* (8), 5538–5542.

(17) Miller, A.; Abrahams, E. Impurity Conduction at Low Concentrations. *Phys. Rev.* **1960**, *120* (3), 745–755.

(18) Köhler, A.; Bässler, H. What Controls Triplet Exciton Transfer in Organic Semiconductors? *J Mater Chem* **2011**, *21* (12), 4003–4011.

(19) Birks, B.; Slifkin, M. A. Pi-Electronic Excitation and Ioniazation Energies of Condensed Ring Aromatic Hydrocarbons. *Nature* **1961**, *191* (4790), 761–764.

(20) Clarke, R. H.; Hochstrasser, R. M. Location and Assignment of the Lowest Triplet State of Perylene. *J. Mol. Spectrosc.* **1969**, *32* (2), 309–319.

(21) Albrecht, W. G.; Michel-Beyerle, M. E.; Yakhot, V. Exciton Fission in Excimer Forming Crystal. Dynamics of an Excimer Build-up in Alpha-Perylene. *Chem. Phys.* **1978**, *35*, 193–200.

(22) Giri, G.; Prodhan, S.; Pati, Y. A.; Ramasesha, S. A Model Exact Study of the Properties of Low-Lying Electronic States of Perylene and Substituted Perylenes. *J. Phys. Chem. A* **2018**, *122* (43), 8650–8658.

(23) Montalti, M.; Credi, A.; Prodi, L.; Gandolfi, M. T. *Handbook of Photochemistry*, 3rd ed.; CRC Press/Taylor and Francis: Boca Raton, 2006.

(24) Carmichael, I.; Hug, G. L. Triplet–Triplet Absorption Spectra of Organic Molecules in Condensed Phases. *J. Phys. Chem. Ref. Data* **1986**, *15* (1), 1–250.

(25) Huang, Z.; Li, X.; Yip, B. D.; Rubalcava, J. M.; Bardeen, C. J.; Tang, M. L. Nanocrystal Size and Quantum Yield in the Upconversion of Green to Violet Light with CdSe and Anthracene Derivatives. *Chem. Mater.* **2015**, *27* (21), 7503–7507.

(26) Xu, Z.; Huang, Z.; Li, C.; Huang, T.; Evangelista, F. A.; Tang, M. L.; Lian, T. Tuning the Quantum Dot (QD)/Mediator Interface for Optimal Efficiency of QD-Sensitized Near-Infrared-to-Visible Photon Upconversion Systems. *ACS Appl. Mater. Interfaces* **2020**, *12* (32), 36558–36567.
